# The impact of participating in basic medical insurance on depression scores of rural middle-aged and older adults—an empirical analysis based on CFPS data

**DOI:** 10.3389/fpubh.2025.1583822

**Published:** 2025-04-25

**Authors:** Ziyin Liao, Rui Zhou, Jingwei Huang, Qing Wang, Jiajing Xu

**Affiliations:** ^1^Dong Fureng Economic and Social Development School, Wuhan University, Wuhan, China; ^2^Department of the Sixth Health Care, The Second Medical Center, Chinese PLA General Hospital, Beijing, China

**Keywords:** basic medical insurance, rural middle-aged and older adults, depression score, bidirectional fixed effect, CFPS

## Abstract

**Introduction:**

According to the latest research by the World Health Organization (WHO), the disease burden caused by depression has risen to the second place in the world, and will rise to the first place by 2030. Currently, there are approximately 90 million individuals with depression in China, with rural middle-aged and older adults facing higher risks due to factors such as weak economic foundations and poor health. This study empirically examines the effect of basic medical insurance in reducing depression scores (measured by the CES-D scale) among rural middle-aged and older adults and validates its underlying mechanisms.

**Methods:**

Using panel data from the China Family Panel Studies (CFPS) in 2012 and 2018, this study constructs a two-way fixed effects model to analyze the relationship between basic medical insurance and depression scores. Heterogeneity analysis was conducted through grouped regression, while robustness checks were performed using panel Probit regression and Quantile regression. Additionally, moderation and mediation effect models were employed to analyze the mechanisms through which basic medical insurance reduces depression scores in this population.

**Results:**

The study finds that basic medical insurance has a positive effect on reducing depression scores among rural middle-aged and older adults. Grouped regression results reveal heterogeneity across subgroups, with weaker improvement effects observed among subgroups aged over 60, females, and those with spouses. By introducing an interaction term between insurance enrollment and chronic disease status into the baseline model, the study identifies a moderating effect of chronic disease on the depression-reducing impact of basic medical insurance. Mediation analysis using the three-step method and bootstrap approach demonstrates that household income per capita partially mediates this effect. Robustness checks support the main findings, and quantile regression indicates that the effect of basic medical insurance is most pronounced among individuals with mild depression or near-threshold depression scores.

**Discussion:**

The research contributes to explaining the dynamic relationship between basic medical insurance and depression among rural middle-aged and older adults, enriching theoretical studies on the impact of basic medical insurance on mental health in this population. The findings hold significant theoretical implications.

## 1 Introduction

The degree and speed of population aging in China are increasing rapidly. Internationally, the standard for an aging society is that the proportion of the population aged 60 and above exceeds 10% (1). Judging by this standard, China entered an aging society long ago. In addition, an important factor affecting population aging is the middle-aged population aged 45 and above. This part of the population has large demographic cohort and will become senior citizens within a decade. As China’s population aging intensifies, addressing the physical and mental health challenges faced by middle-aged and older adults has become an urgent priority. In terms of physical health, with the increase in age, the middle-aged and older adults are more likely to experience the deterioration of physical functions and irreversible damage to various organs. The incidence of chronic diseases such as cardiovascular and cerebrovascular diseases, diabetes, and some malignant tumors increases year by year. In terms of mental health disorders, the middle-aged and older adult population are also more susceptible to psychological diseases such as dementia, depression, and anxiety (2). Against this background, achieving healthy aging has become a critical issue. It is essential to enable middle-aged individuals to transition smoothly into a healthy old age and to help the older adults maintain their physical and mental well-being. These efforts are crucial for reducing the social pension burden and healthcare costs.

According to the latest WHO research, the disease burden associated with depression has risen to second place globally and is projected to rank first by 2030. In China, it is conservatively estimated that approximately 90 million people have depression. Middle-aged and older adults in rural areas are more likely to develop depression due to factors such as weak economic foundations and poor health conditions. The problem of depression not only causes great harm to the mental health of rural middle-aged and older adults, but also indirectly affects their normal physiological functions, exacerbates the deterioration of their physical health, and thus leads to additional consumption of medical resources. Therefore, the direct and indirect losses it brings are substantial, and it is urgent to strengthen the research on the mental health problems of this group.

There may be following main innovations: Firstly, there is a scarcity of using large-sample micro databases to study on the effect of basic medical insurance on depression scores, also the mechanism of the effect of basic medical insurance on depression remains unclear. This study attempts to analyze the mechanism of basic medical insurance in reducing the depression scores of middle-aged and older rural residents by studying the sample data of the same group of middle-aged and older rural residents in 2012 and 2018. This is conducive to explaining the dynamic relationship between basic medical insurance and depression among middle-aged and older rural residents, enriches the theoretical research on the impact of basic medical insurance on the mental health of middle-aged and older rural residents, and thus has certain theoretical significance. In addition, cross-sectional data cannot effectively control individual heterogeneity, making it difficult to achieve unbiased estimation. This paper uses the two-way fixed effects model, which, to a certain extent, overcomes the limitations of cross-sectional data in the time dimension and the disturbances caused by individual heterogeneity.

## 2 Literature review

Theoretically, the health behavior theory and the stress relief pathway provide two theoretical perspectives for understanding how basic medical insurance affects mental health. Health behavior theory provides a valuable framework for understanding how health insurance can influence mental health outcomes. According to the Health Belief Model (HBM), individuals are more likely to engage in preventive health behaviors if they perceive themselves as susceptible to a health problem and believe that the benefits of taking action outweigh the costs (3). In the context of health insurance, this model suggests that insured individuals may be more likely to seek mental health care due to reduced financial barriers and increased perceived benefits of treatment. This aligns with our findings that participation in basic medical insurance is associated with lower depression scores among rural middle-aged and older adults in China. The health behavior theory posits that health insurance can influence mental health through its impact on health - seeking behaviors. For instance, individuals with health insurance are more likely to access preventive care, early diagnosis, and treatment services (4). In the context of rural middle - aged and older adults, access to such services can lead to better management of chronic conditions, which in turn can reduce the risk of developing depression. A study by Smith et al. (5) found that insured individuals were more likely to adhere to medical treatment plans, leading to improved physical health and potentially better mental well - being. This is relevant to our study as rural middle - aged and older adults with basic medical insurance may be more likely to seek help for physical ailments, and better physical health can have a positive spill - over effect on mental health. Another important theoretical perspective is the stress relief pathway. Financial stress is a significant contributor to mental health problems, particularly depression. Health insurance can act as a buffer against financial stress by reducing out-of-pocket expenses for healthcare services. The Conservation of Resources (COR) theory posits that individuals strive to acquire, maintain, and protect their resources, and the loss of resources can lead to stress and negative mental health outcomes (6). By providing financial protection, health insurance can help individuals conserve their resources and reduce stress, thereby improving mental health.

Additionally, both domestic and foreign scholars have conducted relevant studies on the relationship between basic medical insurance and the improvement of depression levels. In Lee et al. (7) study on the relationship between education and the degree of mental depression, it was found that medical insurance could significantly explain the changes in the depression levels of the sample population. In a study of a standard medical insurance plan in Oregon, United States, Finkelstein et al. (8) found that participating in this medical insurance plan could significantly improve the mental health of people aged 19–64. According to the researches from Finkelstein et al. and Baicker et al. (8, 57), the Oregon Health Insurance Experiment (OHIE) represents one of the most rigorous randomized controlled trials (RCTs) in health economics, examining the causal effects of Medicaid expansion on low-income adults in the U.S. Medicaid coverage led to a 30% reduction in depression prevalence and improved self-reported mental health status. This effect was primarily driven by reduced financial strain and increased access to outpatient mental health services and prescription medications. While mental health outcomes improved significantly, OHIE found no statistically meaningful effects on physical health metrics (e.g., blood pressure, cholesterol), underscoring the specificity of insurance effects on psychological well-being. According to UK’s tax-funded NHS provides universal access to mental health services, including the Improving Access to Psychological Therapies (IAPT) program. Chen et al. (9) research on the systems note that reduce socioeconomic disparities in depression treatment, though challenges remain in rural service delivery. Another study investigated the effects of copayments on mental healthcare utilization in the Netherlands. Lambregts et al. (10) found that higher copayments led to a significant decrease in the use of mental health services, particularly among males and individuals with lower socioeconomic status. This highlights the importance of affordable health insurance coverage in promoting mental health utilization and reducing disparities in access to care. In recent years, some domestic scholars have also focused on the impact of medical insurance on mental health and depression scores and have reached different conclusions. In some correlation studies of disease incidence in the field of public health policy, Liliang Long et al. (11) found that in rural areas, older adults who paid for their own medical expenses were more likely to become depressed. Jie Guo et al. (12) used an Ordered Logit Model and found that basic medical insurance could significantly improve physical and mental health, thereby enhancing residents’ sense of fairness. There are also studies that found that basic medical insurance could improve residents’ well-being (13), which contributes to improving their mental health. Changyong Yu et al. (14) incorporated psychological performance into the evaluation system of medical insurance to assess the level of disease-related anxiety and found that medical insurance had an improving effect on the degree of worry about diseases among rural groups, which could reduce their depression scores.

From an economic perspective, Shi Zheng et al. (15), based on a survey of farmers in Jiangsu, Shandong, Anhui, and Henan provinces, found that the New Rural Cooperative Medical System could significantly improve the physical and mental health of rural residents. Pengfei Zhang (16) used the CESD-10 questionnaire score as a proxy variable for mental health and found through empirical analysis that medical insurance could reduce the depression score levels of the older adults by increasing their utilization rate of medical services. In contrast, Qin Zhou et al. (17) used the Charls 2011–2013 data and also used the CES-D questionnaire score as the explained variable to conduct an empirical analysis of whether the New Rural Insurance and the New Rural Cooperative Medical System in social insurance could reduce the depression level. The results showed that the pension insurance had a significant effect on reducing the depression score during the receiving stage, while the effect of the New Rural Cooperative Medical System on score reduction was not obvious. Yaqing Li et al. (18), using mental depression and cognitive health as proxy variables for mental health, found that medical insurance could not only reduce depression scores by promoting the utilization of medical services and improving health levels but also might improve mental health by increasing the safety expectations and life satisfaction of rural middle-aged and older adults. Zhengwen Wang et al. (19), when studying the impact of basic medical insurance on the quality of life of rural middle-aged and older adults, took depression status and life satisfaction as one of the indicators of subjective quality of life and used the principal component analysis method to form a comprehensive evaluation index of quality of life. Tsang et al. (20) found that household income was a mediating factor for basic medical insurance to improve the comprehensive quality of life of middle-aged and older adults in rural areas.

In addition to the mechanisms identified in the above-mentioned literature, per capita household income may also be one of the action paths through which basic medical insurance reduces the depression scores of rural middle-aged and older adults. First of all, several studies have found that basic medical insurance can increase the income level of rural middle-aged and older adults. Liangshu Qi (21), through panel data, with the farmer poverty rate and farmer per capita income as the core explained variables, conducted a multiple regression and found that the New Rural Cooperative Medical System had a significant poverty-reduction and income-increasing effect on low-income and middle-income farmers. Lu Chen and Maomao Xiong (22), in a study of the impact of medical insurance on well-being, also found that participating in basic medical insurance could enhance the well-being of the older adults by increasing the household income level. Jian Zhou et al. (23), by incorporating per capita household income as one of the indicators for measuring poverty into the research, found that basic medical insurance, as one of the tools for income redistribution, could increase their income level and significantly reduce the poverty of the rural older adult population. Some foreign studies have also found that basic medical insurance can improve their health status, increase their employment rate and productivity, and thus increase their household income (24). In addition, basic medical insurance enhances the insured’s ability to resist disease risks. It also allows them to reduce precautionary savings previously allocated for health protection and expenditures. This part of the money can be used for investment in production capital and training of family members’ production skills, thereby increasing the overall household income level (25). In addition, in the literature review in the previous section, several studies have found that the household income level has a significant impact on the depression scores of rural middle-aged and older adults. Therefore, average household income may be one of the potential intermediate mechanisms and requires further empirical testing.

## 3 Theoretical hypotheses

In this paper, two classical theories are applied: the Public Goods Theory and Maslow’s Hierarchy of Needs. The public goods theory was proposed by Samuelson in the 1950s. According to his definition, standard public goods possess the characteristics of non-excludability, non-rivalry, and non-profitability. For basic medical insurance, while following the principle of voluntariness during the insurance-participation stage, it encourages universal participation, and everyone can purchase basic medical insurance to obtain corresponding protection. Therefore, basic medical insurance has non-excludability. At the same time, since the state mainly adopts the pay-as-you-go system when establishing basic medical insurance and does not aim for profit, it has non-profitability. After the formation of the medical insurance fund, when an insured person needs to have a certain proportion of medical expenses reimbursed by basic medical insurance due to illness or other reasons, this part of the fund resources can no longer be used by others. Under the global budget system, in extreme cases, the annual quota of a certain hospital may be exceeded, and the medical expenses within the catalog incurred by a patient when seeking medical treatment near the medical insurance settlement node in this hospital cannot be reimbursed. Therefore, individuals may create a certain degree of rivalry among other insured persons during the reimbursement stage. To sum up, basic medical insurance has the characteristics of quasi-public goods. In domestic research, Xiucai Yin (26) pointed out that medical insurance is a part of social security, with non-excludability and positive externalities, belonging to a type of quasi-public goods, and can only be provided by the government to achieve fair distribution. In addition, some research has found that during the implementation of basic medical insurance, due to differences in the demand and accessibility of medical services among different groups, there may be a reverse redistribution from vulnerable groups to powerful groups, such as the phenomenon of rural residents subsidizing urban residents (27–29), which undoubtedly weakens the protection of basic medical insurance for vulnerable groups (56). Therefore, based on the public goods theory, the government needs to identify the vulnerable groups in basic medical insurance and give appropriate policy tilt. Through policy adjustments, their benefits can be enhanced to achieve the optimal allocation of basic medical insurance, the quasi-public good. Some research also points out that the government should provide more support for public health resources in the mental health of rural older adults (30). Combining with the research theme of this paper, it is necessary to identify the vulnerable groups among rural middle-aged and older adults with high depression scores, conduct an empirical analysis to determine whether the effect of basic medical insurance in reducing their depression scores is weaker than that of other rural middle-aged and older adults, and then provide targeted suggestions based on the current situation.

Maslow’s Hierarchy of Needs Theory is one of the classic theories in psychology. This theory posits that human needs can be divided into five levels from low to high, namely physiological needs, safety needs, Love and belonging needs, esteem needs, and need for self-actualization, with each level ascending in order (31). In Maslow’s theory of needs, only when the lower-level needs are met can the higher-level needs potentially play a motivating role. At the same time, Maslow also pointed out that human mental health, that is, self-actualization, corresponds to reaching the level of self - actualization needs in Maslow’s Hierarchy of Needs Theory. And achieving self-actualization requires the support of physiological needs, safety needs, Love and belonging needs, and esteem needs. Therefore, if basic medical insurance can promote the elevation of the Maslow’s need levels of rural middle-aged and older adults, it can play a role in reducing their depression scores and enhancing their mental health. Specifically, Physiological needs form the foundation of mental health. When people’s physiological needs are met, ensuring they receive enough nutrients and sleep, the body can regulate emotions normally. This helps avoid mood disorders caused by endocrine disruptions and reduces the risk of depression. Safety needs encompass personal safety, economic stability, and emotional security. A stable and secure environment can alleviate internal anxiety and fear, thereby reducing the susceptibility to depression. Love and belonging needs are manifested as a longing for intimacy and a sense of belonging within a group. The frustration of such needs is a common cause of maladjustment and psychological problems. The lack of intimacy and social support can give rise to feelings of loneliness and isolation, which, in the long term, can increase the likelihood of depression. The fulfillment of esteem needs leads to feelings of confidence and self-worth. Lack of recognition and respect from others can make people feel that their value is not reflected, resulting in frustration and a sense of loss, which can easily lead to self-doubt and denial, resulting in low mood and increasing the risk of depression. Self - actualization needs pertain to an individual’s pursuit of realizing their full potential. When an individual is unable to achieve their self - defined goals, the gap between the ideal and the reality can cause a profound sense of frustration and loss. This internal conflict and contradiction have a negative impact on mood, causing the person to fall into a depressive mood.

Empirical evidence has similarly shown that depression is negatively related to need fulfillment (32). This study lent support to Maslow’s perspective that psychological functioning and adaptation are intertwined with the fulfillment of basic needs. Maslow posited that higher-order needs carry greater value compared to lower-order needs. When individuals reach the highest hierarchy of self-actualization needs, they are capable of experiencing an unparalleled sense of fulfillment and satisfaction, and their mental health attains its zenith. It is only when a person’s needs at a particular level are minimally satisfied that they commence to pursue the needs at a higher level, following a hierarchical progression (33). Consequently, an upward shift in Maslow’s hierarchy of needs leads to a substantial enhancement in mental health. This also implies that the fulfillment of Maslow’s needs and the elevation of the need hierarchy can effectively reduce the severity of depression, thus may lowering depression scores.

**Figure 1:**
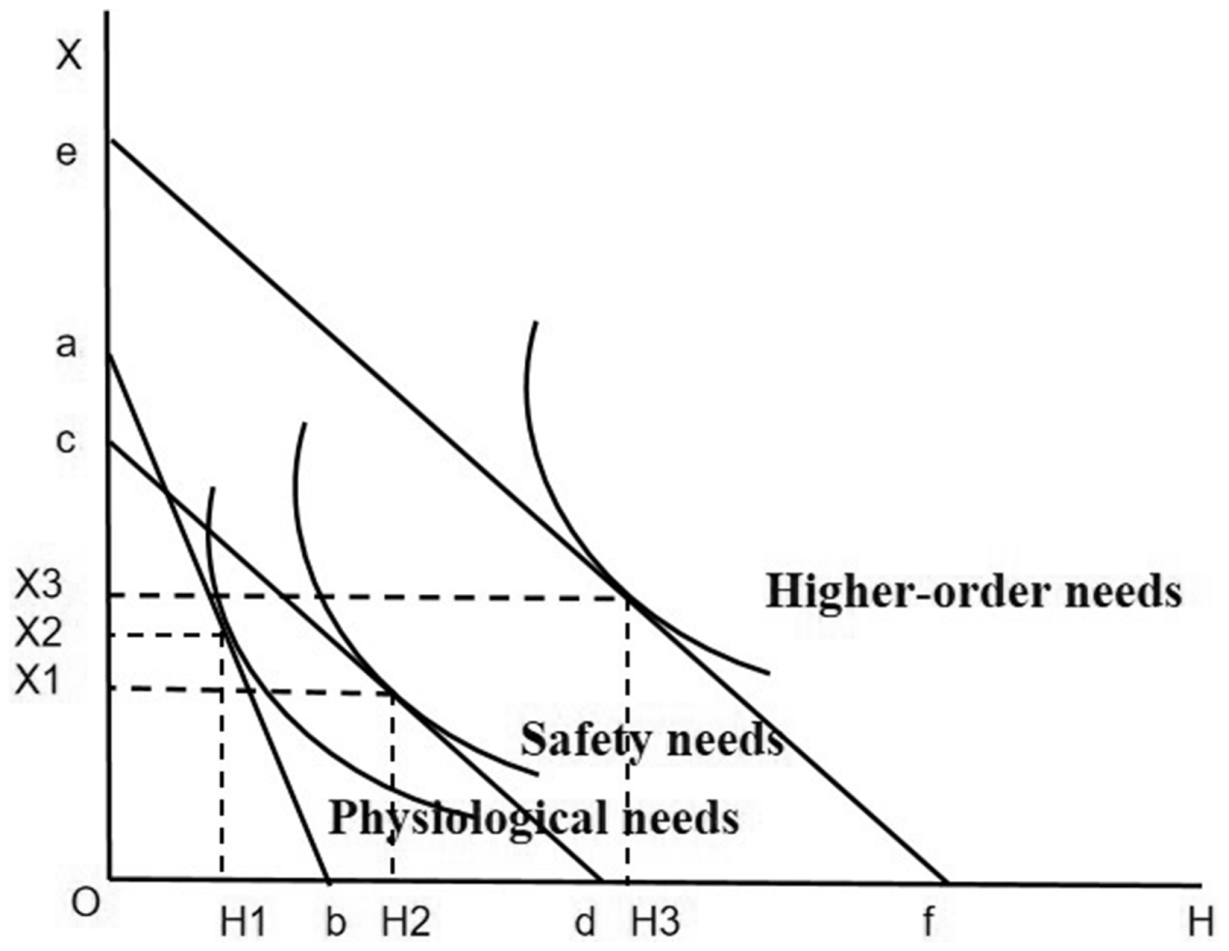
The promotion of Maslow’s needs by basic medical insurance.

To explore how basic medical insurance can enhance the Maslow’s need levels of rural middle-aged and older adults and reduce their depression scores, this study analyzes as follows: Assume that the personal endowments such as time and wealth of rural middle-aged and older adults are fixed. The horizontal axis OH represents an individual’s health level, which can be improved by purchasing medical services and medicines. The vertical axis OX represents a series of other products, including exercise and leisure, social activities, etc., and an increase in the consumption of these products can effectively reduce the depression level of rural middle-aged and older adults (34). Before having basic medical insurance, rural middle-aged and older adults could not get reimbursement for medical services and medicines when seeing a doctor, and it was expensive to maintain health through medical services and medicines. At this time, the budget constraint line was ab. Under the utility maximization strategy, the individual’s physical health condition was H1, with a relatively low health level. Only the physiological needs could be met, while the safety needs could not be guaranteed. After having basic medical insurance, as rural middle-aged and older adults can get a certain degree of medical expense reimbursement, the relative price of improving the health level decreases. At this time, the budget constraint line faced by the individual is cd. Under the utility maximization strategy, the physical health condition is improved to H2, and their Maslow’s needs also rise to the level of safety needs. Finally, as analyzed in the previous section, basic medical insurance may increase the household income per capita of rural middle-aged and older adults. At this time, their budget constraint line becomes ef. Under the utility maximization strategy, the physical health condition is H3, and the consumption of other products is X3. Compared with the situation without basic medical insurance, their health status and Maslow’s need levels have been improved after participating in the insurance, their depression scores have been reduced, and their mental health level has also been correspondingly enhanced (Figure 1).

Based on the comprehensive results of the literature review and the analysis of basic theories, from a public health perspective, basic medical insurance theoretically can play a positive role in reducing the depression scores of rural middle-aged and older adults, which has also been confirmed by some empirical studies in the field of economics. In addition, according to existing literature, by improving household income per capita and ensuring the treatment of the group suffering from chronic diseases, participating in basic medical insurance may also reduce the depression scores of rural middle-aged and older adults. However, whether it is really as described in the theoretical analysis needs to be verified through data and empirical methods to draw a conclusion consistent with the facts. Therefore, to verify whether basic medical insurance actually has the effect of improving the depression scores of rural middle-aged and older adults and whether its mechanism of action exists, this study proposes the following three hypotheses to be tested empirically.

First, according to previous literature research, there is a correlation between the availability of basic medical insurance and the probability of depression among rural middle-aged and older adults. Both at home and abroad, the probability of depression among people with basic medical insurance is lower than that among those without it. However, regarding whether basic medical insurance can reduce the depression scores of rural middle-aged and older adults in the CES-D questionnaire, some studies believe its effect is not obvious. The Public Goods Theory emphasizes that basic medical insurance has the attribute of public goods, which can provide universal medical security for rural middle-aged and older adults, enabling them to obtain economic support and medical services when facing disease risks. This sense of security and protection helps to reduce their anxiety and depressive emotions, thereby lowering the depression score. As a public good, the medical security provided by basic medical insurance can meet the safety needs of rural middle-aged and older adults and enhancing their Maslow’s hierarchy of needs. The improvement of the hierarchy of needs will greatly enhance the mental health level of rural middle-aged and older adults, and correspondingly reduce their scores of the CES-D questionnaire. Therefore, through direct and indirect channels, basic medical insurance should be able to reduce their CES-D questionnaire scores and the probability of depression. Based on the above analysis, the first hypothesis of this paper is proposed:

*H1:* Basic medical insurance can reduce the depression scores of rural middle-aged and older adults.

Second, the effect of basic medical insurance on the depression scores of rural middle-aged and older adults in the CES-D questionnaire may be moderated by whether the older adults themselves suffer from chronic diseases. Suffering from chronic diseases is one of the risk factors for depression among middle - aged and older adults (1). Therefore, rural middle-aged and older adults with chronic diseases generally have higher CES-D questionnaire scores. Facing the same policy, the marginal effect on the mental health of rural middle-aged and older adults with chronic diseases may be more obvious. That is, compared with the healthy middle-aged and older adult group with low depression scores, basic medical insurance may be more likely to reduce the depression scores of the diseased group and thus improve their mental health status. In addition, rural middle-aged and older adults with chronic diseases belong to the vulnerable group in terms of depression. Based on the public goods theory, it is necessary to give policy inclinations to vulnerable groups. In fact, since 1999, China has been actively exploring the inclusion of chronic diseases in the outpatient reimbursement of medical insurance and has achieved certain results (35). At the same time, for the middle-aged and older adults in rural areas with chronic diseases, their physiological needs and safety needs are more urgent due to the disease. Basic medical insurance may play a more prominent role in reducing depression scores of rural middle-aged and older adults, as it can meet their needs to a greater extent and relieve the psychological pressure caused by the disease. Based on the above analysis, the effect of basic medical insurance in reducing the depression scores of rural middle-aged and older adults with chronic diseases may be different from that of healthy rural middle-aged and older adults. Therefore, the second hypothesis of this paper is proposed:

*H2:* The presence of chronic diseases has a moderating effect on the reduction of depression scores of rural middle-aged and older adults by basic medical insurance. Compared with healthy rural middle-aged and older adults, the basic medical insurance has a more significant effect in reducing the depression scores of rural middle-aged and older adults who suffer from chronic diseases.

Finally, basic medical insurance may reduce the scores of rural middle-aged and older adults in the CES-D questionnaire by affecting household income per capita, that is, household income per capita plays a partial mediating role in the mechanism research of this paper. First, according to the Public Goods Theory, basic medical insurance, as part of social security, should have the function of income redistribution. Rural middle-aged and older adults have a poor economic foundation and a low overall welfare level, and should be the objects of subsidy. According to Maslow’s hierarchy of needs theory, when rural middle-aged and older adults participate in basic medical insurance, their income will increase, their budget constraint line will shift outwards, and the increase in income will enable the Maslow’s need levels of rural middle-aged and older residents to be improved and further satisfied, and their mental health status will be improved. In other words, As part of social security, basic health insurance increases the income of rural middle-aged and older adults through income redistribution, which enables them to better satisfy higher hierarchy of needs and thus improve their mental health. In addition, according to the content of the literature review, some studies have shown that household income is the mediating mechanism for basic medical insurance to improve the comprehensive quality of life of rural middle-aged and older adults (19). Empirical evidence also supports the mechanism. Empirical studies have shown that basic medical insurance has an impact on income, and income also affects mental health. Therefore, it can be inferred that per capita household income mediates the relationship between basic medical insurance and depression scores. Specifically, empirical evidence has been shown that basic health insurance can enhance household income. Zhu and Liao (36) used the propensity score matching method to find that basic medical insurance can significantly increase household income, and there is a causal relationship between the two. The health capital of the household plays a mediating role therein. The improvement of health capital can effectively enhance the household’s ability to make a living, improve the efficiency of livelihood activities, and thus increase the level of household income. Secondly, basic health insurance can prevent families from falling into poverty by reducing catastrophic expenditures. Kuroki (37) finds that increases in medical insurance coverage for low-income people in the U.S. reduce medical bankruptcies, and that having health insurance reduces the risk of out-of-pocket medical expenditures, which reduces the likelihood of falling into financial distress. Finally, basic medical insurance has a positive impact on reducing income inequality among older households (38), while rural older households tend to have lower economic status, in this sense basic health insurance can also improve the per capita household income level of rural middle-aged and older adults.

Empirical studies have shown that income is an important influencing factor on mental health. Overall, increases in both personal income and household income can reduce the average experience of negative emotions and the high-frequency negative emotions associated with severe mental illness (SMI), which have a significant positive impact on the mental health of individuals (39). Research on the factors influencing the mental health of older adults has also shown that the higher the level of personal income, the better the mental health of older adults (40). Wen and Cheng (41) found that economic status, represented by annual per capita household income, has a significant impact on the mental health of middle-aged and older adults in rural China, Specifically, for every one thousand yuan increase in annual per capita household income, the CES-D score, which reflects the degree of depression, decreases significantly by 0.293. Therefore, household income per capita may also be a potential mediating mechanism in this study. Therefore, based on the above analysis, the third hypothesis of this paper is proposed:

*H3:* Household income per capita has a mediating effect on the reduction of depression scores of rural middle-aged and older adults by basic medical insurance.

## 4 Methodology

### 4.1 Data

The data employed in this paper is sourced from the China Family Panel Studies (CFPS). The CFPS is meticulously designed to offer real-world tracking and feedback regarding China’s demographic, educational, social, economic, and health transformations. During each survey year, the CFPS undertakes the tracking and collection of data from individuals, families, and communities and generates four sub-databases, namely, the individual database, the children database, the family relationship database, and the family economy database. These databases serve to provide first-hand empirical data for academic research and government decision-making processes. The CFPS project takes the sample population from the 2010 national survey as the baseline sample and a new wave of surveys is carried out biennially, incorporating both the tracking sample from the baseline year and the newly added sample.

We chose the data from 2012 and 2018 for the following rationales: Firstly, regarding data comparability, the CESD20SC variable, which is based on the Center for Epidemiologic Studies Depression Scale (CES-D) scores, was constructed from the adult data published starting from 2016. This variable was derived by standardizing the data from subsequent surveys using the range of depression rating scale scores in 2012. As a result, the depression scores of the sample possess a similar reliability and validity to those of the sample in 2012. Hence, the 2012 data were utilized as the base sample in this study. Secondly, in terms of data timeliness, although the CFPS has released the data of 2020 individual database, due to the requirement of controlling variables in this study, some indicators need to be obtained from the family-relationship database and the family-economy database. Therefore, this study employs the survey data in 2018 and 2012 to form a balanced panel data.

Since this study focus on the influence of basic medical insurance on rural middle-aged and older individuals, the sample is confined to rural middle-aged and older adults who were 45 years old or above in 2012. Those who did not complete the follow-up in 2018 are excluded to acquire the second-period balanced panel data. The final dataset encompasses 5,024 samples with 10,048 observations.

### 4.2 Variables

#### 4.2.1 Dependent variables

The depression score is mainly obtained by integrating the scores of the subjects after they complete the depression scale according to the corresponding statistical rules. The Center for Epidemiologic Studies Depression Scale (CES-D) was developed by Sirodff of the US National Institute of Mental Health in 1977. After several adaptations, it can be divided into multiple versions according to the number of questions. In the data collected by the CFPS project, the 20-item standard version of the CES-D questionnaire and the 8-item short-version questionnaire are mainly used. However, through mathematical processing, the two can be directly compared. When studying economic issues, Qin Zhou et al. (17) also directly use the depression scale score to measure the mental health status of the sample. This study uses the responses to the CES-D depression scale in the database as a scoring tool to measure the depression scores of rural middle-aged and older adults. The reliability and validity of this scale among the middle-aged and older adult population have been repeatedly verified (42, 43).

Referring to the methods of Qin Zhou et al. (17), we use the scores of the Center for Epidemiologic Studies Depression Scale (CES-D) questionnaire as a metric for depression scores. The CFPS 2018 questionnaire uses the 8-question version of CESD8, while the CFPS2012 questionnaire uses CESD20 which contains 20 questions (refer to Appendix Table A1). For instance, the CESD8 questionnaire encompasses the questions presented in Table 1.

**Table 1:** CES-D8 questionnaire questions.

Question no.	Content of the question
1	I’m feeling low.
2	I find it hard to do anything.
3	I do not sleep well.
4	I’m feeling good.
5	I’m feeling lonely.
6	I’m happy with my life.
7	I’m feeling sad.
8	I do not think life can go on.

The CES-D questionnaire assigns an independent score to each question, with a scoring range of 1–4 points per question. Specifically, Questions 1, 2, 3, 5, 7, and 8 are scored positively. That is, the more severe the situation described in these questions, the higher the corresponding score; conversely, Questions 4 and 6 are scored negatively, meaning that the stronger the positive emotion expressed, the lower the score for these questions. Subsequently, aggregate the scores of all questions. A higher total score indicates a more severe state of psychological depression. Through randomized sample assignment and equip percentile equating, when reporting the 2018 data, the CFPS project team constructed a longitudinally comparable variable, CESD20sc, based on the depression scores reported in 2012. In this study, the CES-D20 depression questionnaire scores obtained in 2012 and the percentile-equivalentized CESD20sc scores in 2018 were utilized to construct a novel variable, depression, serving as the explained variable for the empirical analysis. Within the sample of this study, depression spanned a range from 20 to 73 points. Notably, a higher numerical value of this variable indicated a more severe state of depression.

#### 4.2.2 Treatment variable

This research designates whether the sample is covered by basic medical insurance (medsure_dum) as the treatment variable. This variable is represented as a dummy variable. Taking the value of 1, when the sample is enrolled in either the New Rural Cooperative Medical Scheme (NRCMS) or the Urban and Rural Residents’ Basic Medical Insurance (URRBMI), and taking the value of 0, when the sample does not have either type of basic medical insurance.

#### 4.2.3 Control variables

Based on existing studies in the literature, two types of control variables included in CFPS data set were selected to more precisely identify the net effect of basic medical insurance on depression scores. The first type comprises personal-characteristic variables, such as age, gender, the presence of chronic diseases, recent smoking behavior, the presence of a spouse, educational attainment, and self - assessed health status. The second set consists of family-characteristic variables, including the number of children, per capita family income, and the area of residence. Details are presented in Table 2.

**Table 2:** Description of variable names.

Dimension	Variable	Definition	Range of values
Individual characteristic variables	gender	Gender of respondents	0 = female; 1 = male
	age	Age of respondents	[45,100]
	chronic	Whether the respondent has a chronic disease	0 = not having chronic disease; 1 = having chronic disease
	smoke	Whether the respondent has smoked recently or not	0 = not smoked; 1 = smoked
	spouse	Whether the respondent has a spouse or not	0 = not having a spouse; 1 = having a spouse
	edu	Educational level of respondents	0 = illiterate/semi-literate; 1 = elementary school; 2 = middle school; 3 = high school; 4 = College and above
	SAH	Respondents’ self-assessed health status	1 = extremely healthy; 2 = Very healthy; 3 = Relatively healthy; 4 = Fair; 5 = Unhealthy
Household characteristics variables	child_num	Number of children of respondents	[0,9]
	indinc_net	Per capita household income of respondents	Total household income divided by number of household members
	region	Area of residence of respondents	1 = in the Eastern; 2 = in the Central region; 3 = in the Western

### 4.3 Descriptive statistics

**Figure 2:**
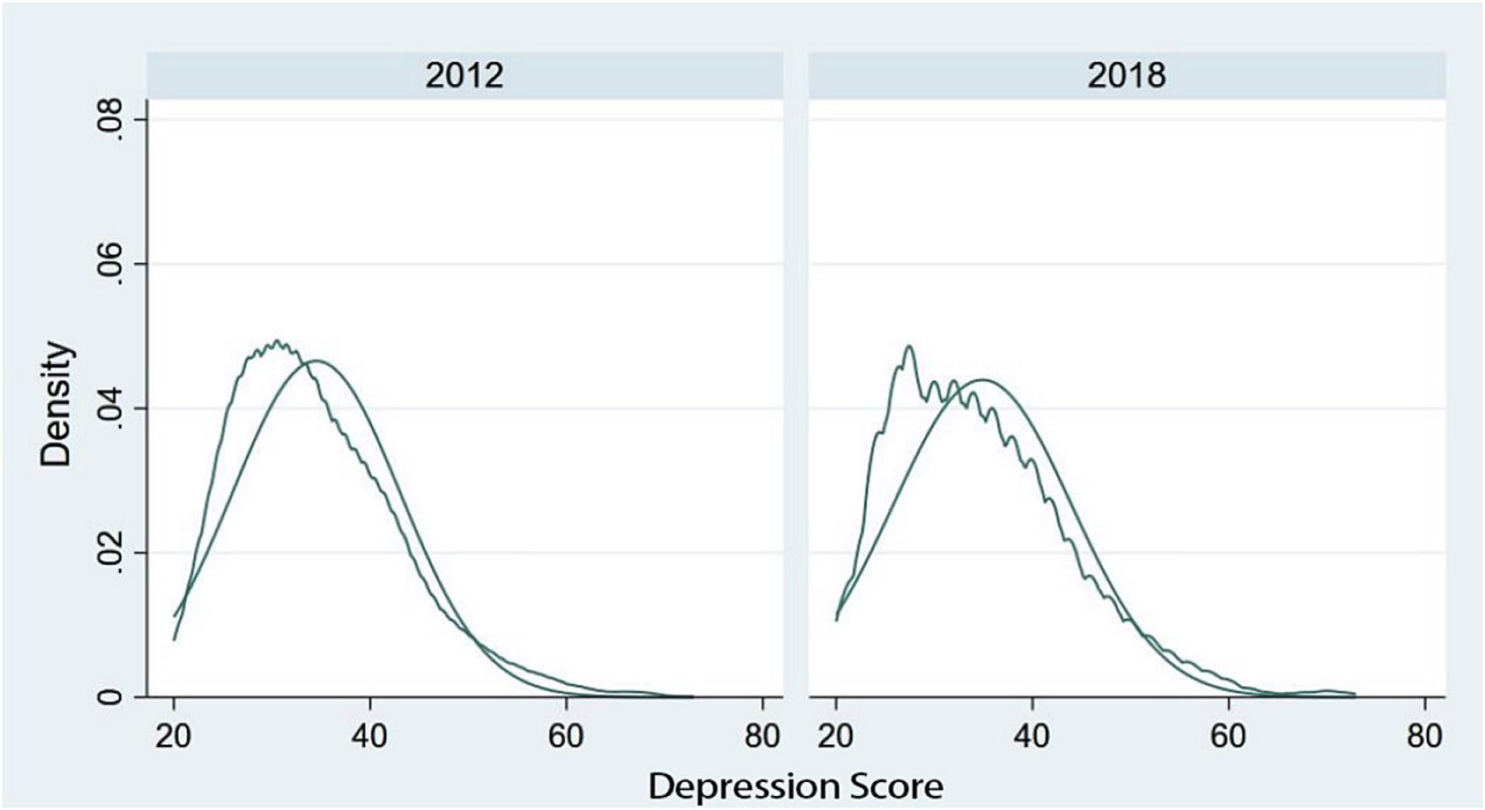
Density distribution of depression scores in the sample.

Figure 2 depicts the density distribution of the depression scores of rural middle-aged and older individuals within the sample. Here, the horizontal axis represents the scores of the CESD questionnaire, while the vertical axis denotes the density of the sample at that specific score within the total sample. As is evident from Figure 2, the distribution of depression scores among rural middle-aged and older adults in 2012 and 2018 exhibited minimal divergence and generally adhered to a normal distribution. Additionally, it can be noted that in both 2012 and 2018, the depression scores in the approximate interval of 50 and above presented a distribution density curve higher than that of the normal distribution. This implies that the proportion of the sample population potentially experiencing depressive emotions might be higher than what is assumed under normal circumstances, which warrants attention. Appendix Tables A2, A3 presents the basic statistics of the rural middle-aged and older adult sample in 2012 and 2018.

Table 3 presents the overall descriptive statistics for the sample in 2012 and 2018. Within the selected sample, the mean depression scores in 2012 and 2018 were nearly identical. However, the standard deviation increased, indicating that there were changes in the scores of the CES-D questionnaire among a part of the middle-aged and older population. Additionally, it is noteworthy that from 2012 to 2018, the per capita annual household income of the sample population underwent significant changes. The mean value increased from 8,498 yuan per year to 11,644 yuan per year. Based on the literature review, the increase in the per capita income of some households may be attributed to their participation in basic health insurance. Moreover, the growth in household income has a positive influence on the mental health of rural middle-aged and older adults. This is the mechanism that requires empirical investigation in this paper.

**Table 3:** Descriptive statistics of the overall sample.

Variable	2012			2018		
	Observation	Mean	Std. Dev.	Observation	Mean	Std. Dev.
depression	5,024	34.51	8.567	5,024	34.90	9.078
medsure_dum	5,024	0.930	0.242	5,024	0.946	0.244
age	5,024	56.29	8.043	5,024	62.28	8.037
SAH	5,024	3.508	1.214	5,024	3.436	1.311
chronic	5,024	0.159	0.365	5,024	0.275	0.446
smoke	5,024	0.347	0.476	5,024	0.329	0.470
gender	5,024	0.513	0.500	5,024	0.513	0.500
spouse	5,024	0.921	0.270	5,024	0.873	0.333
edu	5,024	0.917	1.034	5,024	0.937	1.029
indinc_net	5,024	8,498	9,980	5,024	11,644	13,058
child_num	5,024	2.470	1.167	5,024	1.500	1.596

### 4.4 Identification strategy

#### 4.4.1 Benchmark model

Responses to depression questionnaires are subjective and thus may be affected by the unobservable characteristics of individuals. To mitigate the estimation bias stemming from this unobservable influence, it is essential to control for it to the greatest extent possible. Simultaneously, given that this study employs balanced panel data and the explained variable is a continuous score whose distribution density function can be approximately in line with the normal distribution, both the fixed-effect model and the random-effect model can be utilized for regression. However, the choice between the two models requires a Hausman test for model specification. Moreover, this paper reports the mixed ordinary least squares (OLS) regression of the 2012 and 2018 data as a reference, and the results can serve as a form of reference for robustness checks. The fixed-effect model is specified as in Equation (1):


(1) $$\mathrm{depressio}n_{\mathrm{it}}=\lambda_{i}+\gamma_{t}+\beta_{1}\mathrm{medsure}\_dum_{\mathrm{it}}+\beta X+\varepsilon_{\mathrm{it}}$$

Where ${\mathrm{depression}}_{\mathrm{it}}$ is the depression score of individual i at period t, $\lambda_{i}$ denotes the individual-specific fixed effect of individual i that remains invariant over time, $\gamma_{t}$ is the time-fixed effect of period t, $\beta_{1}$ is the coefficient of the dummy variable for the presence of basic medical insurance, X is a set of control variables, and $\varepsilon_{\mathrm{it}}$ is the random disturbance term. According to the research content of this paper, the main concern is to examine the magnitude of the $\beta_{1}$ in the regression results and its significance level. If $\beta_{1}$ is negative in sign and statistically significant, it implies that basic medical insurance has the effect of reducing the scores on the CES-D questionnaire among the rural middle-aged and older population. The coefficient of interest is $\beta_{1}$ , which captures the impact of basic medical insurance on the depression scores of rural middle-aged and older individuals.

#### 4.4.2 Moderating effect model

In the descriptive statistical analysis, a significant increase in the number of rural middle-aged and older individuals suffering from chronic diseases with the increase in age was observed. Then, the question arises: does the presence of chronic diseases influence the reducing effect of basic medical insurance on the depression scores of rural middle-aged and older adults? The moderating-effect model is employed to explore whether the impact of the explanatory variables on the explained variable varies according to different values of the moderator variable, which can be a categorical variable. In this study, the moderating effect of chronic diseases is empirically analyzed by incorporating the variable of whether an individual has a chronic disease, as well as the interaction term between the variable of whether one has basic medical insurance and the variable of whether one has chronic disease, into the baseline model. Subsequently, the regression equation is transformed into Equation (2):


(2) $$\begin{array}{l} \mathrm{depressio}n_{\mathrm{it}}=\lambda_{t}+\gamma_{t}+{\beta_{1}}^{\prime }\mathrm{medsure}\_dum_{\mathrm{it}}+\beta_{2}\mathrm{chronic}+\\ \beta_{3}\mathrm{chronic}\times \mathrm{medsure}\_dum+\beta X+\varepsilon_{\mathrm{it}} \end{array}$$

Where, β1’ represents the coefficient of the impact of participating in basic medical insurance on the sample’s depression score after isolating the influence of chronic diseases, β2 is the coefficient of the impact of having a chronic disease on the depression score, and β3 is the coefficient of the impact on the depression score when having basic medical insurance in the presence of a chronic disease.

#### 4.4.3 Mediating effect model

**Figure 3:**
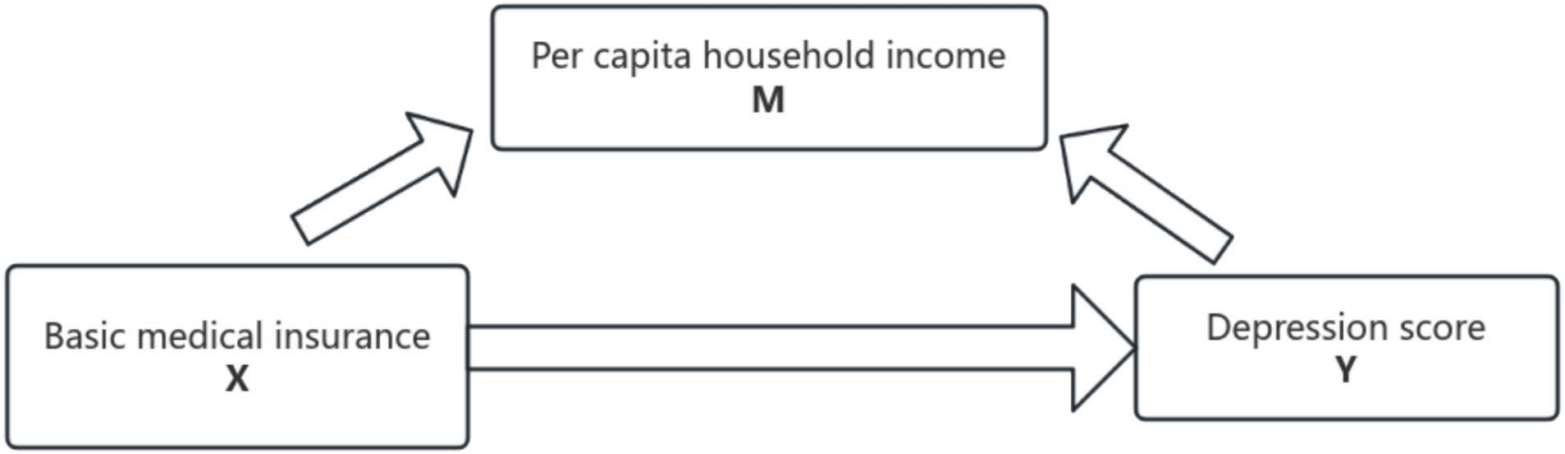
Mediating-effect model.

Based on the literature review and theoretical analysis, per capita household income might serve as a mediator variable in the process through which basic medical insurance reduces the depression scores of rural middle-aged and older adult individuals. The mediation effect can be classified into two types: the partial mediating effect and the full mediating effect. A full mediating effect implies that the explanatory variable can influence the explained variable solely via the mediator variable. A partial mediating effect, on the other hand, indicates that the explanatory variable can either directly impact the explained variable or affect the explained variable by acting on the mediator variable. In this paper, in accordance with the framework proposed by Baron and Kenny, we adopt the empirical model based on step-by-step regression put forward by Wen et al. (44) as a validation approach to explore the potential mediating effect of household per capita income. The mechanism of the possible mediating effect is illustrated in Figure 3.

The mediating effect is tested according to the three-step method with the following Equations 3–5:


(3) $$Y=cX+e_{1}$$

(4) $$M=\mathrm{aX}+e_{2}$$

(5) $$Y=c'X+bM+e_{3}$$

**Figure 4:**
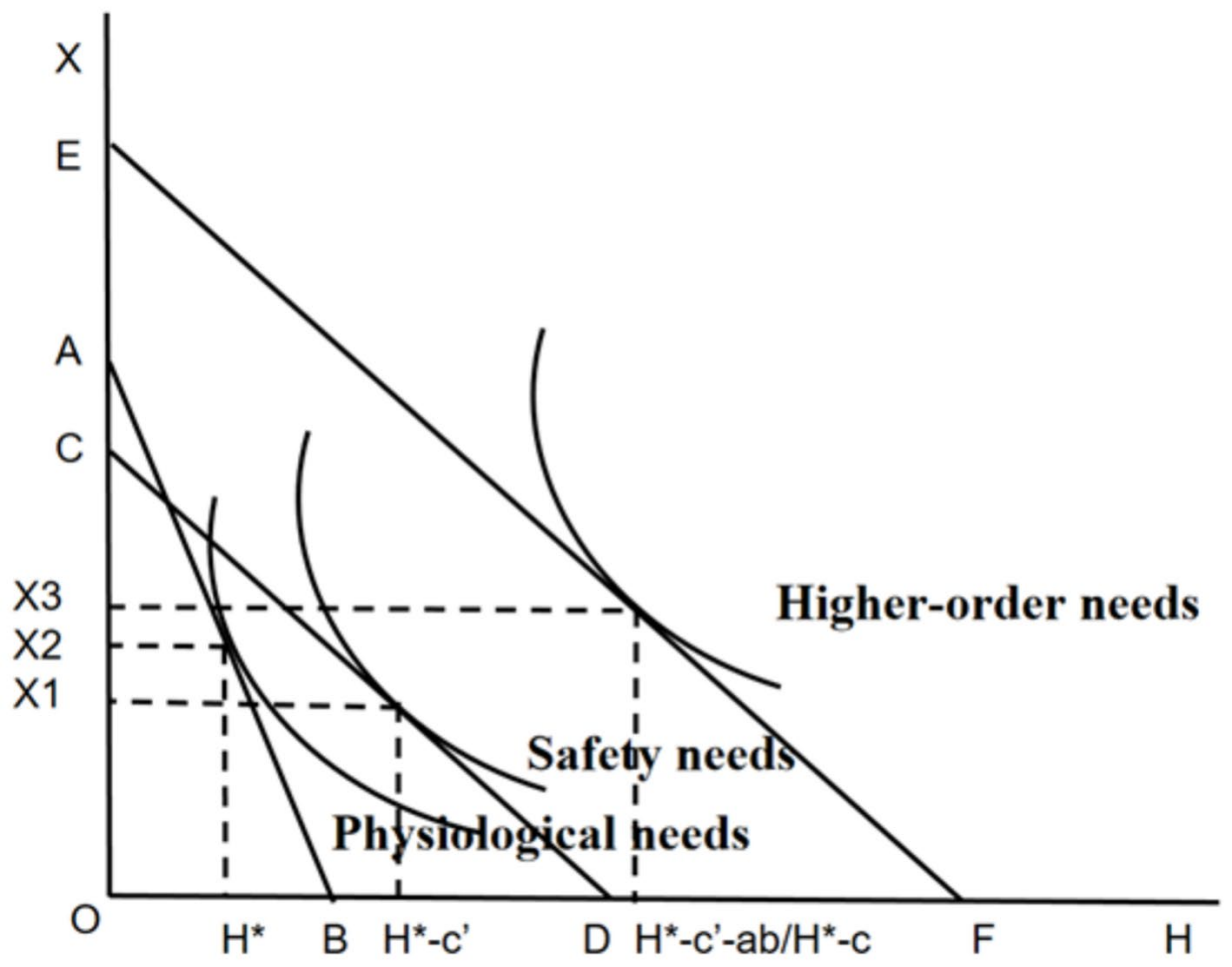
The mediating effect model of basic medical insurance based on Maslow’s hierarchy of needs.

The coefficient c represents the impact of the core explanatory variable, namely the presence or absence of basic medical insurance, on the explained variable, which is the depression score. It reflects the total effect when the mediator variable, per capita household income, is not incorporated into the model. Coefficient a denotes the direct effect of the core explanatory variable (the presence or absence of basic medical insurance) on per capita household income. Coefficient b indicates the explanatory power of the mediator variable M (per capita household income) when it is included in the overall regression. Coefficient c’ represents the direct explanatory effect of having basic medical insurance on depression scores after the decomposition of the mediating effect. These three regressions are carried out in sequence. If the coefficients a, b,c and c’ are all statistically significant, a partial mediation effect exists. If the coefficients a, b, and c are all significant while c’ is not, a full mediation effect is present. In the case where the coefficient c is significant but either a or b is not significant, a bootstrap test is employed. If 0 is not within the 95% confidence interval of the bootstrap results, the mediating effect is significant; otherwise, there is no mediating effect. Figure 4 shows the results of placing the coefficients of the three regression equations into Maslow’s Hierarchy of Needs.

## 5 Results

### 5.1 Baseline results

The regression results of the OLS, fixed-effect, and random-effect models on the 2012 and 2018 balanced panel data are presented in Table 4. It is evident that the coefficient of the variable indicating the presence or absence of basic medical insurance is significant at the 1% level in all three regressions. This finding strongly suggests that having basic medical insurance exerts a distinct effect in reducing the depression scores within the sample population. Possible rationales are as follows. Regarding the direct effect, the possession of basic medical insurance implies that rural middle-aged and older adult individuals can receive a certain proportion of reimbursement for hospitalization and medical expenses when afflicted with major diseases. Consequently, it alleviates their expected economic burden, enhances the sense of security and protection for healthy rural middle-aged and older adults, and mitigates their concerns about potentially exorbitant future medical treatment costs, thereby reducing their depression scores. In terms of the indirect effect, after enrolling in basic medical insurance, rural middle-aged and older adult individuals attach greater importance to their own health conditions and adjust their health-related behaviors. Moreover, when suffering from chronic diseases, having basic medical insurance lowers the cost threshold for treatment and ensures sustained medical treatment, which may indirectly improve their depressive states. Specifically, in the results reported by the OLS model, having basic medical insurance leads to a 1.226-point reduction in depression scores, significant at the 1% level. In the random-effect model, having basic medical insurance results in a 1.171-point reduction in depression scores, also significant at the 1% level. In the results of the fixed-effect model, enrollment in basic medical insurance brings about a 1.009-point reduction in depression scores, significant at the 1% level. Thus, the null hypothesis H1 holds true. A Hausman test is conducted on the fixed-effect model and the random-effect model. The test coefficient is 166.4, significant at the 1% level. As a result, the null hypothesis is rejected, and the fixed-effect model is selected as the baseline reporting model. In the fixed-effect model, the coefficient of the gender variable is omitted due to the control of fixed effects. However, in both the mixed OLS regression and the random-effect regression, the coefficient of gender is significantly negative, indicating that females have significantly higher depression scores than males, which is consistent with the conclusion drawn by Lei et al. (45). Furthermore, it can be observed that having a spouse significantly and substantially reduces the value of depression scores at the 1% level. On average, the depression scores of those with a spouse are 3.566 points lower than those without a spouse. Therefore, the spouseless population is at a disadvantage in terms of depression scores, an aspect that warrants attention. Finally, when comparing the regression coefficients of other control variables, although the coefficient of medsure_dum is significant at the 1% level, its value is relatively small. This may be attributed to the relatively low level of the New Rural Cooperative Medical Scheme, which has a wide coverage in rural areas. Additionally, basic medical insurance is more focused on the reimbursement of hospitalization expenses and the protection of major diseases after the onset of illness, resulting in a weaker direct impact on the psychological aspect. Based on the above-mentioned analysis, basic medical insurance can be enhanced to maintain the mental health of rural older individuals and reduce their depression scores.

**Table 4:** Results of baseline regression.

	(1)	(2)	(3)
Explanatory variable	Mixed ols	Panel random effects	Panel fixed effects
medsure_dum	−1.226***	−1.171***	−1.009**
	(−3.74)	(−3.69)	(−2.46)
gender	−1.934***	−1.980***	-
	(−9.36)	(−8.62)	-
age	−0.013	−0.003	0.052*
	(−1.22)	(−0.25)	(1.83)
smoke	0.419**	0.378*	0.144
	(2.01)	(1.71)	(0.35)
child_num	−0.106*	−0.118**	0.007
	(−1.79)	(−2.05)	(0.06)
spouse	−3.606***	−3.607***	−3.566***
	(−13.25)	(−12.36)	(−6.08)
edu	−0.804***	−0.822***	−0.611
	(−9.48)	(−8.57)	(−0.71)
Ln(indinc_net)	−0.360***	−0.270***	−0.026
	(−6.02)	(−4.60)	(−0.33)
SAH	2.142***	1.940***	1.153***
	(33.53)	(29.88)	(11.88)
region	−1.005***	−1.022***	−0.404
	(−15.82)	(−14.16)	(−0.27)
Observation	10,048	10,048	10,048
Hausman Test			166.41***
Random Effect		Yes	
Fixed Effect			Yes

Significance levels: *p < 0.1, **p < 0.05, ***p < 0.01. Standard deviation statistics are in parentheses.

### 5.2 Heterogeneity analysis

To comprehensively explore the influence of basic medical insurance on the reduction of depression scores among rural middle-aged and older individuals with diverse individual characteristics, this research conducts heterogeneity analyses from the perspectives of age, gender, and whether or not one has a spouse. The subsequent sections present the detailed findings of these analyses.

#### 5.2.1 Heterogeneity analysis by age

In the exploration of age-based heterogeneity, the sample was partitioned in accordance with whether the individuals were 60 years old in 2012. This categorization was principally grounded in the World Health Organization (WHO) criteria for differentiating middle-aged and older adults. Based on the empirical results, in the subgroup of individuals younger than 60, the reduction in depression scores attributable to the possession of basic medical insurance amounts to 1.005 points, and this effect is statistically significant at the 10% level. In contrast, in the subgroup of those older than 60, the impact of basic medical insurance on reducing depression scores is relatively minor and does not achieve statistical significance. This divergence can be attributed to the following reasons. In the younger-age subgroup, as individuals are in the process of transitioning out of the labor market, the insured ones exhibit less concern about future catastrophic medical expenditures compared to the uninsured, which results in lower depression scores. Conversely, as the older population ages, a multiplicity of health issues gradually surface, and pre-existing chronic conditions are likely to deteriorate. When medical insurance reimbursement effectively reduces the relative price of healthcare services, it stimulates an increase in the healthcare demand of the older adults, as verified by the research of Hu et al. (46). The augmented healthcare demand incurs additional corresponding healthcare expenditures for the older adults. This may heighten their financial stress and influence their spending in other aspects. As a result, it potentially exerts an impact on depression scores, thereby rendering the results for the older subgroup statistically nonsignificant (Table 5).

**Table 5:** Results of heterogeneity analysis by age.

Explanatory variable	(1)	(2)
	<60	>60
medsure_dum	−1.005*	−0.615
	(−1.88)	(−0.83)
control variables	YES	YES
Observation	6,226	2,898
Fixed Effect	Yes	Yes

Significance levels: *p < 0.1, **p < 0.05, ***p < 0.01. Standard deviation statistics are in parentheses.

#### 5.2.2 Heterogeneity analysis by gender

In the baseline regression, it can be observed that the coefficient of gender in the rural middle-aged and older adult population is negative. This implies that women are more likely to have higher depression scores. Then, the question arises: does having basic medical insurance exert the same effect across different gender groups? The empirical results are as follows: In the female group, the explanatory power of basic medical insurance for the depression score is not significant, and the absolute value of the coefficient is relatively small, standing at 0.602. In the male group, the coefficient of having basic medical insurance is significant, and having basic medical insurance can reduce the depression score by 1.453. Similar findings were reported by Li et al. (18) when they analyzed gender heterogeneity using the 2015 cross-sectional data from the China Health and Retirement Longitudinal Study (CHARLS). A possible explanation is that, compared with the female group, the male group allocates more resources in terms of spending and investment on their health after retirement (47). Consequently, the male group not only receives more financial compensation from medical insurance but also derives more mental-health benefits (Table 6).

**Table 6:** Results of heterogeneity analysis by gender.

	(1)	(2)
Explanatory variable	Female	Male
medsure_dum	−0.602	−1.453***
	(−1.01)	(−2.63)
control variables	YES	YES
Observation	4,892	5,156
Fixed Effect	Yes	Yes

Significance levels: *p < 0.1, **p < 0.05, ***p < 0.01. Standard deviation statistics are in parentheses.

#### 5.2.3 Heterogeneity analysis by spouse

In the heterogeneity analysis with respect to the presence or absence of a spouse, it is discernible that in the sample of individuals with a spouse, having basic medical insurance leads to a 0.752-point reduction in depression scores, and this effect is significant at the 10% level. In the non-spouse group, being insured reduces depression scores by 2.086, yet it fails to reach statistical significance. A plausible explanation for this lies in the fact that the sample size of the spouseless population is relatively small, resulting in a limited variance in insurance coverage status. As a consequence, it becomes arduous to obtain a precise estimate. Judging from the regression coefficients, the impact of basic medical insurance participation on the improvement of depression scores for the spouseless population is markedly higher than that for the spousal population. This is likely because the spouseless population generally has a higher baseline level of depression scores. Thus, having basic medical insurance can confer a greater marginal psychological benefit to them, and the effect of reducing their depression scores is more prominent compared to that of the spousal population (Table 7).

**Table 7:** Results of heterogeneity analysis by spouse.

	(1)	(2)
Explanatory variable	Having spouse	Not having spouse
medsure_dum	−0.752*	−2.086
	(−1.71)	(−1.41)
control variables	YES	YES
Observation	8,744	768
Fixed Effect	Yes	Yes

Significance levels: *p < 0.1, **p < 0.05, ***p < 0.01. Standard deviation statistics are in parentheses.

### 5.3 Mechanism

#### 5.3.1 Analysis of the moderating effects of chronic diseases

In the baseline model, an interaction term between the presence of basic medical insurance and the existence of chronic diseases is incorporated to validate the moderating effect of chronic diseases. If the coefficient of the interaction term is statistically significant, hypothesis H2 is deemed to hold. Based on empirical regression analysis, at the 1% significance level, the presence of chronic diseases is found to increase the depression scores of rural middle-aged and older adult individuals by 2.143 points, suggesting that the group with chronic diseases is at a relative disadvantage in terms of depression scores. Moreover, the coefficient of the interaction term between chronic diseases and basic medical insurance is −0.672, which is significant at the 10% level. This finding verifies that chronic diseases exert a moderating effect on the role of basic medical insurance in reducing the depression scores of rural middle-aged and older adults. Furthermore, upon the inclusion of the dummy variable for chronic diseases and the interaction term, the coefficient of medsure_dum changes from −1.015 to −0.829. This alteration reflects the actual impact of basic medical insurance on reducing depression scores. In summary, by interpreting the empirical results of the moderating effect, it can be concluded that chronic diseases have a moderating effect on the role of basic medical insurance in reducing the depression scores of rural middle-aged and older adults. Thus, the null hypothesis H2 is established (Table 8).

**Table 8:** Results of moderating effects analysis of chronic diseases.

	(1)	(2)
Explanatory variable	Baseline regression	Adding an interaction term to regression
chronic	–	2.143**
	–	(2.21)
chronic*medsure_dum	–	−0.672*
	–	(−1.70)
medsure_dum	−1.015**	−0.829*
	(−2.47)	(−1.81)
control variables	Yes	Yes
Observation	10,048	10,048
Fixed Effect	Yes	Yes

Significance levels: *p < 0.1, **p < 0.05, ***p < 0.01. Standard deviation statistics are in parentheses.

#### 5.3.2 Analysis of the mediating effect of per capita household income

The analysis of the mediating effect of per capita household income adheres to the procedures specified in the modeling section 3.2.3. Initially, a three-step regression is carried out, and the regression results are presented as follows. It can be discerned that the coefficient c is significant at the 1% level, signifying that the possession of basic medical insurance exerts an influence in reducing the depression scores of rural middle-aged and older adult individuals. The coefficient a is both significant and positive, demonstrating that having basic medical insurance significantly elevates per capita household income. The coefficient c’ is significant at the 1% level, indicating that even after accounting for the variations in per capita household income, having medical insurance remains effective in reducing depression scores. However, the coefficient b is not significant, in which case, according to the mediation effect test procedure put forward by Wen et al. (44), the bootstrap test should be proceeded with to further ascertain the validity of the mediation effect (Table 9).

**Table 9:** Results of mediating effect analysis of per capita household income.

	(1)	(2)	(3)
Explanatory variable	Depression score	Ln(Indinc_Net)	Depression score
ln(indinc_net)	–	–	−0.026
	–	–	(−0.33)
medsure_dum	−1.014**	0.189*	−1.009**
	(−2.47)	(2.58)	(−2.46)
control variables	Yes	Yes	Yes
Fixed Effect	Yes	Yes	Yes

Significance levels: *p < 0.1, **p < 0.05, ***p < 0.01. Standard deviation statistics are in parentheses.

The results of the bootstrap test are presented in Table 10. Given that 0 lies outside the 95% confidence interval of the indirect-effect test result, the null hypothesis H3 holds. That is, per capita household income can serve as a mediator to partially account for the decrease in the depression scores of the rural middle-aged and older adults group brought about by the possession of basic medical insurance.

**Table 10:** Results of bootstrap test.

	Coefficient	Bootstrap standard error	z-value	*p*-value	Confidence Interval	
_bs_1(indirect effect)	−0.0510986	0.0215971	−2.37	0.018	−0.0934282	−0.008769
_bs_2(direct effect)	−1.059557	0.5171603	−2.05	0.040	−2.073173	−0.0459417

The mediating effect of per capita household income, in conjunction with relevant literature, can potentially be elucidated as follows. Rural middle-aged and older individuals with basic medical insurance enjoy stronger economic security during illness compared to the uninsured group. Their household income is less impacted by illnesses, and they experience lower levels of mental stress, thereby resulting in lower depression scores. Moreover, as the New Rural Cooperative Medical System (NRCMS) and the Urban and Rural Resident Basic Medical Insurance (URRBMI) in some regions are paid on a per-family basis, both the older adults and the main labor force in rural areas are covered. This may enhance per capita household income by alleviating the main labor force’s concerns regarding the health of their family members, enabling them to better commit to their work or select higher-paying workplaces. Higher per capita household income leads to lower depression scores among rural older adults, as they have less anxiety about their future lives and enjoy higher levels of life satisfaction.

## 6 Robustness

### 6.1 Replacing the explanatory variable

The explanatory variable was replaced with a binary variable indicating whether or not one is depressed, and a panel probit model was employed to conduct robustness tests. As a robustness test for the baseline regression in this paper, the updated Radloff’s (48) approach was adopted. Specifically, the 95th percentile of the CES-D20sc indicator in the CFPS dataset, which is 52 points, was used as the criterion for determining whether the rural middle-aged and older adults in the sample were depressed. First, a depression dummy variable (depression_dum) was created with an initial value of 0. If the CES-D scale score of a sample individual exceeded 52, the individual was considered to be in a state of depression, and the depression dummy variable was coded as 1, thereby defining the depression state of the sample. Subsequently, the binary variable of whether depressed was tested through panel probit model regression (Table 11).

**Table 11:** Panel probit regression results.

	(1)
Explanatory variable	Panel probit regression
medsure_dum	−0.261**
	(−2.60)
control variables	YES
Observation	10,048

Significance levels: *p < 0.1, **p < 0.05, ***p < 0.01. Standard deviation statistics are in parentheses.

From the regression results of the probit model, it is evident that rural middle-aged and older individuals covered by medical insurance are significantly less likely to experience depression compared to those without insurance. This empirical finding serves to confirm the robustness of the previous baseline regression.

### 6.2 Quantile regression

Table 12 displays the quantile regression results regarding the impact of basic medical insurance on the depression scores of rural middle-aged and older individuals. These results indicate that the possession of basic medical insurance significantly decreases the depression scores of rural middle-aged and older adults at the 30th and 90th percentile. Concretely, at the 30th percentile of the CES-D score, having basic medical insurance leads to a 0.728-point reduction in depression scores, and at the 90th percentile of the CES-D score, having basic medical insurance reduces depression scores by 1.7 points. For the 10th, 50th and 70th percentile of depression scores, the reduction in CES-D scores attributable to basic medical insurance was not statistically significant. Overall, the regression results across all percentile illustrate that basic medical insurance can lower depression scores. The most pronounced effects are observed at lower levels of depression and the critical depression score. This further underscores the necessity for potentially depressed rural middle-aged and older adult populations to participate in basic medical insurance. It also substantiates the robustness of the conclusion that basic medical insurance can mitigate depression among rural middle-aged and older adults.

**Table 12:** Quantile regression results.

	(1)	(2)	(3)	(4)	(5)
Explanatory variable	QR = 0.1	QR = 0.3	QR = 0.5	QR = 0.7	QR = 0.9
medsure_dum	−0.606	−0.728*	−0.221	−0.547	−1.703**
	(−1.64)	(−1.77)	(−0.54)	(−1.08)	(−2.32)
control variables	YES	YES	YES	YES	YES
Observation	10,048	10,048	10,048	10,048	10,048

Significance levels: *p < 0.1, **p < 0.05, ***p < 0.01. Standard deviation statistics are in parentheses.

## 7 Research conclusion

Using data from the China Family Panel Studies, this paper focuses on middle-aged and older adult individuals in rural areas, aiming to explore how basic medical insurance improves their depression scores. By constructing a balanced panel with the data from 2012 and 2018, after proposing hypotheses based on basic theories, this paper controls for nine variables at the family and individual levels as much as possible and uses econometric methods such as the individual fixed effects model and the mediation model to conduct an overall assessment, heterogeneity analysis, and mechanism test of the improvement of the depression scores of middle-aged and older adults in rural areas by basic medical insurance. The main conclusions are as follows:

(1) Firstly, descriptive statistics and density distribution analysis reveal that depression scores among rural middle-aged and older adult individuals generally follow a normal distribution but exhibit a fat-tailed phenomenon, indicating a higher proportion of individuals with elevated depression scores. In addition, during the research time span, more older adults in the sample population suffered from chronic diseases and spousal bereavement. These may be some of the risk events that lead to an increase in their depression scores.
(2) Secondly, after conducting the pooled OLS model, random effects model, and fixed effects model, it is found that having basic medical insurance has a significant effect on reducing the depression scores of middle-aged and older adults in rural areas, but the improvement is relatively small. This may be because the basic medical insurance covering rural areas mainly includes the New Rural Cooperative Medical System and the Basic Medical Insurance for Urban and Rural Residents, which has been piloted and integrated in various cities since 2016. Their compensation ratios are relatively low, and the reimbursable scope is relatively small, so they cannot significantly improve the sense of security and protection of rural middle-aged and older adults. In addition, when examining the regression coefficients of the control variables, it is found that the depression scores of women and widowed groups increase significantly, verifying their status as vulnerable groups from the perspective of depression.
(3) In the heterogeneity analysis of the sample of rural middle-aged and older adults, the analysis is mainly carried out from three aspects: age, gender, and marital status. The conclusion is that compared with the older adults over 60 years old, having basic medical insurance has a more significant effect on improving the depression scores of middle-aged people in rural areas, and the improvement range is also larger. This may be because middle-aged people in rural areas are at the stage of withdrawing from the labor market. Participating in basic medical insurance can relieve their worries about large-amount medical expenses caused by future illnesses and improve their psychological state. For the older adults, some studies have shown that participating in basic medical insurance will stimulate their medical needs, leading to an increase in their medical expenses, which may bring them an economic burden and weaken the effect of basic medical insurance on improving their depression scores.

In the analysis of gender heterogeneity, this study finds that compared with men, the improvement effect of having basic medical insurance on the depression scores of rural middle-aged and older adult women is not significant, and the absolute value is also smaller than that of men. A possible explanation is that men invest more in health and consume more after retirement, have more opportunities to use medical insurance, thus obtaining more economic compensation from medical insurance and also gaining spiritual benefits. The following will explain why explain why men might invest more in health and consume more after retirement through two key mechanisms: gender income gaps leading to inequality in health opportunities and heterogeneous health impacts of retirement across genders. First, from Keynes’ Absolute Income Hypothesis and Relative Income Hypothesis to the Life-Cycle Hypothesis and Permanent Income Hypothesis, economic theories consistently identify income as the most critical determinant of consumption. Thus, the disparity in post-retirement health investments and expenditures between men and women may stem from gender income gaps. Persistent gender income disparities, driven by factors like human capital differences, occupational segregation, and gender discrimination, remain a global challenge. In the United Kingdom, women’s state pension income is 21% lower than men’s. In China, the gap is even starker: men’s pension income is approximately 1.9 times that of women, as shown by CHARLS 2015 data (49). Second, retirement, as a pivotal life-course transition, involves the loss of social roles and economic stability, posing adaptive challenges that may adversely affect physical and mental health. Studies consistently reveal gender differences in retirement’s health impacts. For instance, using an ecological life-course model, Kim and Moen (50) found that retirement initially improved men’s positive attitudes toward aging but increased depressive symptoms long-term, with no significant effects for women. In China, Lei et al. (51) analyzed national census data and concluded that normal-age retirement negatively impacted men’s health but had no effect on women. Conversely, Liu et al. (52) utilized CFPS data to show that retirement significantly improved women’s self-rated health—attributed to increased exercise frequency post-retirement—while men’s health remained unaffected.

In summary, scholarly consensus indicates that retirement exerts gender-heterogeneous health effects, disproportionately harming men’s well-being. This may drive men to prioritize health investments and consumption post-retirement as a compensatory mechanism.

In the heterogeneity analysis of marital status, although it is found through coefficient comparison that the marginal effect of having basic medical insurance on improving depression is greater for those without a spouse than for those with a spouse, the result is not significant due to the sample size.

(4) Finally, in the mechanism test of the impact of basic medical insurance on the depression scores of rural middle-aged and older adults, the moderating effect of chronic diseases and the mediating effect of household income per capita are tested. The empirical results show that suffering from chronic diseases has a moderating effect on the reduction of the depression scores of rural middle-aged and older adults by participating in insurance. At the same time, the depression scores of people suffering from chronic diseases are also higher than those of ordinary rural middle-aged and older adults, making them one of the vulnerable groups. In the test of the mediating effect of household income per capita, the three-step method and the bootstrap method are used, and the results show that household income per capita has a partial mediating effect. In general, chronic disease status plays a moderating role in the relationship between rural middle-aged and older adult people’s participation in basic medical insurance and depression score. Older adults represent a high-risk population for chronic diseases. In China, 75.23% of older adults live with chronic conditions, while the prevalence of depression in this demographic has risen substantially (53). Both domestic and international studies demonstrate a strong correlation between depressive states and chronic diseases, with older chronic disease patients exhibiting significantly higher rates of depression compared to the general older adult population (54). Notably, rural older adults with chronic diseases experience higher depression rates than their urban counterparts. This disparity is likely attributable to urban–rural gaps in social security coverage and economic resources, which contribute to divergent psychological outcomes (55). The introduction of basic medical insurance has mitigated disparities in social security and economic resources for older adults with chronic diseases, bolstering their life confidence. Consequently, chronic disease status serves as a moderating factor in the relationship between participation in basic medical insurance and reduced depression scores among rural middle-aged and older adults.

## 8 Implications for policy and practice

Through an analytical approach that combines theory and empirical evidence, this paper demonstrates that basic medical insurance can improve depression scores and enhance mental health status among rural middle-aged and older adults. However, at the current stage, there is a gap in reimbursement levels between the Urban and Rural Residents’ Basic Medical Insurance (URRBMI) and other forms of basic medical insurance. The coverage for outpatient services and medications is limited, and the improvement in depression scores among vulnerable groups is not significant. In response to these issues, this paper puts forward the following recommendations from the perspectives of policy formulation and healthcare practice:

From the perspective of policy formulation: (1) While basic medical insurance has played a positive role, its effectiveness is limited due to the low level of medical insurance coverage in rural areas. Mental health problems among rural middle-aged and older adults are more severe. Since the integration of NCMS and URBMI, the situation has improved, but there is still a gap between the actual reimbursement ratio and the policy target. Therefore, it is necessary to further enhance the medical insurance coverage for rural middle-aged and older adults, such as increasing the reimbursement for hospitalization expenses and determining the reimbursement ratio based on income. (2) Basic medical insurance has attributes of welfare and redistribution and should emphasize fairness. In terms of depression scores, there are differences among rural middle-aged and older adults. Women, widowed individuals, and those with chronic diseases may belong to vulnerable groups. In this regard, appropriate preferential treatment should be given to these groups in terms of medical insurance coverage.

From the perspective of medical practice: (1) expand the catalog of medications and medical services, and include more outpatient items and commonly used medications. This will allow rural middle-aged and older adults to benefit from the treatment of minor illnesses and improve their depression scores. (2) Population aging has led to an increase in the prevalence of chronic diseases among rural middle-aged and older adult individuals. The high treatment costs of chronic diseases impose a double burden on both medical insurance funds and patients, affecting their mental health. Basic medical insurance should follow the principle of “prevention first, combining prevention and treatment,” strengthen pre-disease prevention, compensate for the preventive medical expenses of high-risk groups for chronic diseases, and improve the three-level prevention system for chronic diseases. Through this approach, the burden on medical insurance can be reduced, the sense of gain among the people can be enhanced, and depression scores can be lowered. (3) For rural middle-aged and older adults who already suffer from chronic diseases, regular free clinics, examinations, and psychological counseling should be conducted, and subsidies for medication and care expenses should be provided. This will slow down the progression of their diseases and promote their mental health.

## Data Availability

Publicly available datasets were analyzed in this study. This data can be found at: https://www.isss.pku.edu.cn/cfps/en/.
