# Supplementary material for: The impact of participating in basic medical insurance on depression scores of rural middle-aged and older adults—an empirical analysis based on CFPS data
Source: Front Public Health. 2025 Apr 25;13:1583822. doi: 10.3389/fpubh.2025.1583822 (PMC12061694; doi:10.3389/fpubh.2025.1583822)
Supplement: Supplementary file 1 [file Table_1.docx]

**Appendix**

**Table A1.** CES-D20 questionnaire questions

| Question No. | Content of the question |
| --- | --- |
| 1 | I get upset over little things. |
| 2 | I don't want to eat. I have a bad appetite. |
| 3 | Even with the help of my family and friends, I couldn't get rid of my bitterness. |
| 4 | I don't think I'm as good as most people. |
| 5 | I can't concentrate when I'm doing things. |
| 6 | I'm feeling down. |
| 7 | I feel like I'm struggling to do anything. |
| 8 | I feel hopeless about the future. |
| 9 | I feel like my life is a failure. |
| 10 | I'm scared. |
| 11 | I'm not sleeping well. |
| 12 | I'm not happy. |
| 13 | I'm talking less than usual. |
| 14 | I'm feeling lonely. |
| 15 | I feel that people are not very friendly to me. |
| 16 | I don't think life is fun. |
| 17 | I used to cry. |
| 18 | I feel sad. |
| 19 | I feel like people don't like me. |
| 20 | I don't think I can go on with my life. |

**Table A2.** Basic situation of middle-aged and elderly people in rural areas in 2012

| Year | Variable | Variable Description | Min | Max | Frequency | Percentage |
| --- | --- | --- | --- | --- | --- | --- |
| 2012 | depression | Depression Score | 20 | 73 | - | - |
|  | medsure_dum | Having Basic Medical Insurance | 0 | 1 | 4672 | 0.93 |
|  |  | Not Having Basic Medical Insurance |  |  | 352 | 0.07 |
|  | age | - | 45 | 86 |  |  |
|  | SAH | Extremely Healthy | 0 | 5 | 311 | 0.062 |
|  |  | Very Healthy |  |  | 698 | 0.139 |
|  |  | Relatively Healthy |  |  | 1587 | 0.316 |
|  |  | Fair |  |  | 985 | 0.196 |
|  |  | Unhealthy |  |  | 1443 | 0.287 |
|  | chronic | Having Chronic Disease | 0 | 1 | 797 | 0.159 |
|  |  | Not Having Chronic Disease |  |  | 4227 | 0.841 |
|  | smoke | Smoked | 0 | 1 | 1744 | 0.347 |
|  |  | Not Smoked |  |  | 3280 | 0.653 |
|  | gender | Male | 0 | 1 | 2578 | 0.513 |
|  |  | Female |  |  | 2446 | 0.487 |
|  | spouse | Having A Spouse | 0 | 1 | 4626 | 0.921 |
|  |  | Not Having A Spouse |  |  | 398 | 0.079 |
|  | edu | Illiterate/Semi-Literate | 0 | 4 | 2388 | 0.475 |
|  |  | Elementary School |  |  | 1149 | 0.229 |
|  |  | Middle School |  |  | 1035 | 0.206 |
|  |  | High School |  |  | 418 | 0.083 |
|  |  | College And Above | 1 |  | 34 | 0.007 |
|  | indinc_net |  | 0 | 270467 |  |  |
|  | child_num |  | 0 | 9 |  |  |
|  | region | In The Eastern Region | 1 | 3 | 1865 | 0.371 |
|  |  | In The Central Region |  |  | 1492 | 0.297 |
|  |  | In The Western Region |  |  | 1667 | 0.332 |

**Table A3.** Basic situation of middle-aged and elderly people in rural areas in 2018

| Year | Variable | Variable Description | Min | Max | Frequency | Percentage |
| --- | --- | --- | --- | --- | --- | --- |
| 2018 | depression | Depression Score | 22 | 72 |  | - |
|  | medsure_dum | Having Basic Medical Insurance | 0 | 1 | 4753 | 0.946 |
|  |  | Not Having Basic Medical Insurance |  |  | 271 | 0.054 |
|  | age | - | 50 | 92 |  | - |
|  | SAH | Extremely Healthy | 0 | 5 | 568 | 0.113 |
|  |  | Very Healthy |  |  | 471 | 0.094 |
|  |  | Fairly Healthy |  |  | 1715 | 0.341 |
|  |  | Average |  |  | 743 | 0.148 |
|  |  | Unhealthy |  |  | 1527 | 0.304 |
|  | chronic | Having Chronic Disease | 0 | 1 | 1380 | 0.275 |
|  |  | Not Having Chronic Disease |  |  | 4227 | 0.725 |
|  | smoke | Smoked | 0 | 1 | 1653 | 0.329 |
|  |  | Not Smoked |  |  | 3280 | 0.671 |
|  | gender | Male | 0 | 1 | 2578 | 0.513 |
|  |  | Female |  |  | 2446 | 0.487 |
|  | spouse | Having A Spouse | 0 | 1 | 4386 | 0.873 |
|  |  | Not Having A Spouse |  |  | 638 | 0.127 |
|  | edu | Illiterate/Semi-Literate | 0 | 4 | 2304 | 0.459 |
|  |  | Elementary School |  |  | 1224 | 0.244 |
|  |  | Middle School |  |  | 1042 | 0.207 |
|  |  | High School |  |  | 418 | 0.083 |
|  |  | College And Above |  |  | 36 | 0.007 |
|  | indinc_net |  | 0 | 286500 | - | - |
|  | child_num |  | 0 | 9 | - | - |
|  | region | In The Eastern Region | 1 | 3 | 1864 | 0.371 |
|  |  | In The Central Region |  |  | 1490 | 0.297 |
|  |  | In The Western Region |  |  | 1670 | 0.332 |
